# A qualitative study identifying implementation strategies using the i-PARIHS framework to increase access to pre-exposure prophylaxis at federally qualified health centers in Mississippi

**DOI:** 10.1186/s43058-024-00632-6

**Published:** 2024-08-28

**Authors:** Trisha Arnold, Laura Whiteley, Kayla K. Giorlando, Andrew P. Barnett, Ariana M. Albanese, Avery Leigland, Courtney Sims-Gomillia, A. Rani Elwy, Precious Patrick Edet, Demetra M. Lewis, James B. Brock, Larry K. Brown

**Affiliations:** 1 Department of Psychiatry, Rhode Island Hospital, One Hoppin Street, Coro West, 204, Providence, RI 02903 USA; 2 Department of Psychiatry and Human Behavior, Warren Alpert Medical School of Brown University, Providence, RI USA; 3 Department of Population Health Science, University of Mississippi Medical Center, Jackson, MS USA; 4 Department of Medicine, University of Mississippi Medical Center, Jackson, MS USA; 5 Center for Healthcare Organization and Implementation Research, VA Bedford Healthcare System, Bedford, MA USA

**Keywords:** HIV Prevention, Implementation Science, Pre-exposure prophylaxis (PrEP), Telemedicine

## Abstract

**Background:**

Mississippi (MS) experiences disproportionally high rates of new HIV infections and limited availability of pre-exposure prophylaxis (PrEP). Federally Qualified Health Centers (FQHCs) are poised to increase access to PrEP. However, little is known about the implementation strategies needed to successfully integrate PrEP services into FQHCs in MS.

**Purpose:**

The study had two objectives: identify barriers and facilitators to PrEP use and to develop tailored implementation strategies for FQHCs.

**Methods:**

Semi-structured interviews were conducted with 19 staff and 17 PrEP-eligible patients in MS FQHCs between April 2021 and March 2022. The interview was guided by the integrated-Promoting Action on Research Implementation in Health Services (i-PARIHS) framework which covered PrEP facilitators and barriers. Interviews were coded according to the i-PARIHS domains of context, innovation, and recipients, followed by thematic analysis of these codes. Identified implementation strategies were presented to 9 FQHC staff for feedback.

**Results:**

Data suggested that PrEP use at FQHCs is influenced by patient and clinic staff knowledge with higher levels of knowledge reflecting more PrEP use. Perceived side effects are the most significant barrier to PrEP use for patients, but participants also identified several other barriers including low HIV risk perception and untrained providers. Despite these barriers, patients also expressed a strong motivation to protect themselves, their partners, and their communities from HIV. Implementation strategies included education and provider training which were perceived as acceptable and appropriate.

**Conclusions:**

Though patients are motivated to increase protection against HIV, multiple barriers threaten uptake of PrEP within FQHCs in MS. Educating patients and providers, as well as training providers, are promising implementation strategies to overcome these barriers.

Contributions to the literature

- We propose utilizing Federally Qualified Health Centers (FQHCs) to increase pre-exposure prophylaxis (PrEP) use among people living in Mississippi.
- Little is currently known about how to distribute PrEP at FQHCs.
- We comprehensively describe the barriers and facilitators to implementing PrEP at FQHCs.
- Utilizing effective implementation strategies of PrEP, such as education and provider training at FQHCs, may increase PrEP use and decrease new HIV infections.

## Introduction

The HIV outbreak in Mississippi (MS) is among the most critical in the United States (U.S.). It is distinguished by significant inequalities, a considerable prevalence of HIV in remote areas, and low levels of HIV medical care participation and virologic suppression [1]. MS has consistently ranked among the states with the highest HIV rates in the U.S. This includes being the 6th highest in new HIV diagnoses [2] and 2nd highest in HIV diagnoses among men who have sex with men (MSM) compared to other states [2–4]. Throughout MS, the HIV epidemic disproportionately affects racial and ethnic minority groups, particularly among Black individuals. A spatial epidemiology and statistical modeling study completed in MS identified HIV hot spots in the MS Delta region, Southern MS, and in greater Jackson, including surrounding rural counties [5]. Black race and urban location were positively associated with HIV clusters. This disparity is often driven by the complex interplay of social, economic, and structural factors, including poverty, limited access to healthcare, and stigma [5].

Pre-exposure prophylaxis (PrEP) has gained significant recognition due to its safety and effectiveness in preventing HIV transmission when taken as prescribed [6–9]. However, despite the progression in PrEP and its accessibility, its uptake has been slow among individuals at high risk of contracting HIV, particularly in Southern states such as MS [10–14]. According to the CDC [5], *“4,530 Mississippians at high risk for HIV could potentially benefit from PrEP, but only 927 were prescribed PrEP.”* Several barriers hinder PrEP use in MS including limited access to healthcare, cost, stigma, and medical mistrust [15–17].

Federally qualified health centers (FQHCs) are primary healthcare organizations that are community-based and patient-directed, serve geographically and demographically diverse patients with limited access to medical care, and provide care regardless of a patient’s ability to pay [18]. FQHCs in these areas exhibit reluctance in prescribing or counseling patients regarding PrEP, primarily because they lack the required training and expertise [19–21]. Physicians in academic medical centers are more likely to prescribe PrEP compared to those in community settings [22]. Furthermore, providers at FQHCs may exhibit less familiarity with conducting HIV risk assessments, express concerns regarding potential side effects of PrEP, and have mixed feelings about prescribing it [23, 24]. Task shifting might also be needed as some FQHCs may lack sufficient physician support to manage all aspects of PrEP care. Tailored strategies and approaches are necessary for FQHCs to effectively navigate the many challenges that threaten their patients’ access to and utilization of PrEP.

The main objectives of this study were to identify the barriers and facilitators to PrEP use and to develop tailored implementation strategies for FQHCs providing PrEP. To service these objectives, this study had three specific aims. Aim 1 involved conducting a qualitative formative evaluation guided by the integrated-Promoting Action on Research Implementation in Health Services (i-PARIHS) framework- with FQHC staff and PrEP-eligible patients across three FQHCs in MS [25]. Interviews covered each of the three i-PARIHS domains: context, innovation, and recipients. These interviews sought to identify barriers and facilitators to implementing PrEP. Aim 2 involved using interview data to select and tailor implementation strategies from the Expert Recommendations for Implementing Change (ERIC) project [26] (e.g., provider training) and methods (e.g., telemedicine, PrEP navigators) for the FQHCs. Aim 3 was to member-check the selected implementation strategies and further refine these if necessary. Data from all three aims are presented below. The standards for reporting qualitative research (SRQR) checklist was used to improve the transparency of reporting this qualitative study [27].

## Methods

### Formative evaluation interviews

#### Sample

Interviews were conducted with 19 staff and 17 PrEP-eligible patients from three FQHCs in Jackson, Canton, and Clarksdale, Mississippi. Staff were eligible to participate if they were English-speaking and employed by their organization for at least a year. Eligibility criteria for patients included: 1) English speaking, 2) aged 18 years or older, 3) a present or prior patient at the FQHC, 4) HIV negative, and 5) currently taking PrEP or reported any one of the following factors that may indicate an increased risk for HIV: in the past year, having unprotected sex with more than one person with unknown (or positive) HIV status, testing positive for a sexually transmitted infection (STI) (syphilis, gonorrhea, or chlamydia), or using injection drugs.

#### Data collection

The institutional review boards of the affiliated hospitals approved this study prior to data collection. An employee at each FQHC acted as a study contact and assisted with recruitment. The contacts advertised the study through word-of-mouth to coworkers and relayed the contact information of those interested to research staff. Patients were informed about the study from FQHC employees and flyers while visiting the FQHC for HIV testing. Those interested filled out consent-to-contact forms, which were securely and electronically sent to research staff. Potential participants were then contacted by a research assistant, screened for eligibility, electronically consented via DocuSign (a HIPAA-compliant signature capturing program), then scheduled for an interview. Interviews occurred remotely over Zoom, a HIPAA-compliant, video conferencing platform. Interviews were conducted until data saturation was reached. In addition to the interview, all participants were asked to complete a short demographics survey via REDCap, a HIPAA-compliant, online, data collection tool. Each participant received a $100 gift card for their time.

The i-PARIHS framework guided interview content and was used to create a semi-structured interview guide [28]. Within the i-PARIHS framework’s elements, the interview guide content included facilitators and barriers to PrEP use at the FQHC: 1) the innovation, (PrEP), such as its degree of fit with existing practices and values at FQHCs; 2) the recipients (individuals presenting to FQHCs), such as their PrEP awareness, barriers to receiving PrEP such as motivation, resources, support, and personal PrEP experiences; and 3) the context of the setting (FQHCs), such as clinic staff PrEP awareness, barriers providing PrEP services, and recommendations regarding PrEP care. Interviews specifically asked about the use of telemedicine, various methods for expanding PrEP knowledge for both patients and providers (e.g., social media, advertisements, community events/seminars), and location of services (e.g., mobile clinics, gyms, annual health checkups, health fairs). Staff and patients were asked the same interview questions. Data were reviewed and analyzed iteratively throughout data collection, and interview guides were adapted as needed.

#### Data analysis

Interviews were all audio-recorded, then transcribed by an outside, HIPAA-certified transcription company. Transcriptions were reviewed for accuracy by the research staff who conducted the interviews.

Seven members of the research team (TA, LW, KKG, AB, CSG, AL, LKB) independently coded the transcripts using an a priori coding schedule that was developed using the i-PARIHS and previous studies [15–17]. All research team members were trained in qualitative methods prior to beginning the coding process. The coding scheme covered: patient PrEP awareness, clinic staff PrEP awareness, barriers to receiving PrEP services, barriers to providing PrEP services, and motivation to take PrEP. Each coder read each line of text and identified if any of the codes from the a priori coding framework were potentially at play in each piece of text. Double coding was permitted when applicable. New codes were created and defined when a piece of text from transcripts represented a new important idea. Codes were categorized according to alignment with i-PARIHS constructs. To ensure intercoder reliability, the first 50% of the interviews were coded by two researchers. Team meetings were regularly held to discuss coding discrepancies (to reach a consensus). Coded data were organized using NVivo software (Version 12). Data were deductively analyzed using reflexive thematic analysis, a six-step process for analyzing and reporting qualitative data, to determine themes relevant to selecting appropriate implementation strategies to increase PrEP use at FQHCs in MS [29]. The resulting thematic categories were used to select ERIC implementation strategies [26]. Elements for each strategy were then operationalized and the mechanism of change for each strategy was hypothesized [30, 31]. Mechanisms define how an implementation strategy will have an effect [30, 31]. We used the identified determinants to hypothesize the mechanism of change for each strategy.

### Member checking focus groups

Member checking is when the data or results are presented back to the participants, who provide feedback [32] to check for accuracy [33] and improve the validity of the data [34]. This process helps reduce the possibility of misrepresentation of the data [35]. Member checking was completed with clinic staff rather than patients because the focus was on identifying strategies to implement PrEP in the FQHCs.

#### Sample

Two focus groups were conducted with nine staff from the three FQHCs in MS. Eligibility criteria were the same as above. A combination of previously interviewed staff and non-interviewed staff were recruited. Staff members were a mix of medical (e.g., nurses, patient navigators, social workers) and non-medical (e.g., administrative assistant, branding officer) personnel. Focus group one had six participants and focus group two had three participants. The goal was for focus group participants to comprise half of staff members who had previously been interviewed and half of non-interviewed staff.

#### Data collection

Participants were recruited and compensated via the same methods as above. All participants electronically consented via DocuSign, and then were scheduled for a focus group. Focus groups occurred remotely over Zoom. Focus groups were conducted until data saturation was reached and no new information surfaced. The goal of the focus groups was to member-check results from the interviews and assess the feasibility and acceptability of selected implementation strategies. PowerPoint slides with the results and implementation strategies written in lay terms were shared with the participants, which is a suggested technique to use in member checking [33]. Participants were asked to provide feedback on each slide.

#### Data analysis

Focus groups were all audio-recorded, then transcribed. Transcriptions were reviewed for accuracy by the research staff who completed focus groups. Findings from the focus groups were synthesized using rapid qualitative analyses [36, 37]. Facilitators (TA, PPE) both took notes during the focus groups of the primary findings. Notes were then compared during team meetings and results were finalized. Results obtained from previous findings of the interviews and i-PARIHS framework were presented. To ensure the reliability of results, an additional team member (KKG) read the transcripts to verify the primary findings and selected supportive quotes for each theme. Team meetings were regularly held to discuss the results.

## Results

### Formative evaluation interviews

#### Sample

Thirty-six semi-structured interviews in HIV hot spots were completed between April 2021 and March 2022. Among the 19 FQHC staff, most staff members had several years of experience working with those at risk for HIV. Staff members were a mix of medical (e.g., doctors, nurses, CNAs, social workers) and non-medical (e.g., receptionists, case managers) personnel. Table 1 provides the demographic characteristics for the 19 FQHC clinic staff and 17 FQHC patients.

**Table 1:** Demographic Characteristics of Clinic Staff and PrEP-Eligible FQHC Patients

***Clinic Staff Demographic Characteristics (N* = *19)***	
	**n (%)**
Gender	
Male	18 (95%)
Female	1 (5%)
Race/ethnicity	
Black / African American	18 (95%)
Other	1 (5%)
Mean Years of Experience	9.16 (*SD*: 7.65)
Job Role	
Medical	7 (37%)
Nonmedical	12 (63%)
***PrEP-Eligible FQHC Patient Demographic Characteristics (N* = *17)***	
Mean Age	38.63 (14.92)
	**n (%)**
Gender	
Male	5 (33%)
Female	9 (60%)
Other	1 (7%)
Race/ethnicity	
Black / African American	15 (100%)
Education level	
Some high school	3 (20%)
High school diploma or GED	2 (13%)
Some college, no degree	6 (40%)
Bachelor’s or associate degree	4 (27%)
Health insurance type	
Private	8 (53%)
Public	7 (47%)
Annual household income	
Less than $10,000	5 (33%)
$10,000 to $19,999	3 (20%)
$20,000 to $29,999	1 (7%)
$30,000 to $39,999	3 (20%)
$40,000 to $49,999	3 (20%)
Currently taking PrEP	6 (40%)
Previously used telemedicine	9 (56%)

Note for Patient Demographic Data

N’s varied as below due to missing data

^a^N = 8

^b^N = 15

^c^N = 16

#### Themes

Table 2 provides a detailed description of the findings within each category: PrEP knowledge, PrEP barriers, and PrEP motivation. Themes are described in detail, with representative quotes, below. Implementation determinants are specific factors that influence implementation outcomes and can be barriers or facilitators. Table 3 highlights which implementation determinants can increase ( +) or decrease (-) the implementation of PrEP at FQHCs in MS. Each determinant, mapped to its corresponding i-PARIHS construct, is discussed in more detail below. There were no significant differences in responses across the three FQHCs.

**Table 2:** PrEP knowledge and barriers

Category	Implementation Determinant	Implementation Determinant Information ( ±)	i-PARIHS
***PrEP* Knowledge**	Patient PrEP Awareness	❖ Obtained general PrEP awareness **( +)** ❖ Lacked specific knowledge pertaining to indications, availability, variety of medications, and efficacy** (-)** ❖ Learned about PrEP from commercials, medical providers, and social networks **( +)**	Recipients
	Clinic Staff PrEP Awareness	❖ Varied PrEP knowledge and training **( ±)** ❖ Had PrEP providers and navigators **( +)**	Context
***PrEP Barriers***	Barriers Receiving PrEP Services	❖ Negative side effects **(-)** ❖ Stigma / Insufficient confidentiality **(-)** ❖ Burden of taking a daily pill **(-)** ❖ Low perceived risk of HIV **(-)** ❖ Inadequate transportation **(-)** ❖ Cost of PrEP **(-)**	Recipients Innovation
	Barriers Providing PrEP Services	❖ Lack of PrEP training **(-)** ❖ Discomfort discussing or prescribing PrEP **(-)** ❖ Lack of variety in the kind of providers who offer PrEP (e.g., OB/GYN, primary care) **(-)**	Context
***PrEP Motivation***	Motivation to take PrEP	❖ Protect themselves and their partners **( +)** ❖ Experienced an HIV scare **( +)** ❖ Heard about how PrEP can help them based on their community identity **( +)**	Recipients

**Table 3:** Strategies to Increase PrEP Use in FQHCs

i-PARIHS Component	Them^pe	Implementation Barrier/Determinant ( ±)	ERIC Strategy	Specific Strategy Elements	Mechanism of Strategies
***Strategies Related to PrEP Information Dissemination***					
**Recipients:** Knowledge, Education, Motivations, Values, & Beliefs	PrEP Education Outreach Recommendations	PrEP uninformed /concerned about the side effects of PrEP (-)	Conduct educational meetings Distribute educational materials	❖ PrEP education at schools/colleges ❖ Health fairs, seminars, and interactive outreach webinars ❖ Flyers and pamphlets ❖ HIV testing events ❖ Utilize trusted sources (i.e., pastors and churches) ❖ Social media pages and radio stations ❖ Offer PrEP education during culturally relevant events	Conduct educational meetings in the community about HIV risk and PrEP side effects to increase awareness of the need for PrEP Collaborate with trusted members of the community to disseminate PrEP education to increase PrEP acceptance Distribute PrEP educational materials on social media platforms and radio stations to increase overall awareness of PrEP
**Supportive Quotes**	⇒ *“There are so many people who could benefit from it [PrEP] and don't know about it. Education and awareness is key.”*—Staff, Community Health Worker, Female ⇒ *“They [FQHCs] have to utilize all resources to get their name out. We know social media is at an all-time high. Everybody uses it. Have someone post let’em know [PrEP is available].” -*Patient, Age 50, Male, Taking PrEP ⇒ *“Just a general health fair with havin’ somethin’ about the PrEP services there because, I’m gonna be honest with ya. If it was just a HIV health fair, I probably wouldn’t go.”—*Patient, Age Unknown, Gender Unknown, Not on PrEP ⇒ *“Try to talk about it [PrEP] more, schools, churches, everywhere.” -*Patient, Age Unknown, Female, Taking PrEP ⇒ *“Taking groups in the school. If you start early, it helps prevent, as they’re gettin’ older.”*—Staff, Accountant, Female				
**Recipients:** Knowledge, Education **Context:** Culture **Innovation:** Degree of novelty, Patient experience	Normalize PrEP	Unaware PrEP is for anyone at risk for HIV (-)	Develop educational materials Involve patients/consumers and family members	❖ Create diverse/inclusive advertisements for PrEP ❖ Provide education at a variety of places ❖ Share patient PrEP testimonials ❖ Screen all patients for PrEP ❖ Include PrEP when discussing other prevention concepts	Develop PrEP educational materials that include a variety of ages, races, and gender identities to enhance awareness that anyone at risk for HIV could benefit from PrEP Recruit and train community members who have taken PrEP to share their PrEP testimonials and PrEP education to increase PrEP knowledge and acceptance in the community
**Supportive Quotes**	⇒* “I think that they could possibly find a way for people that are taking PrEP to share their stories about PrEP. That’ll make people feel more comfortable with taking it. If I know some people that’s takin’ it [PrEP], and they feel really secure with it and comfortable, that may encourage me to try PrEP.” -*Patient, Age 30, Female, Not on PrEP ⇒* “I feel like PrEP is for everybody regardless of who you’re sleepin’ with. I can be a churchgoin’ woman and love my husband. Anything can happen. We takin’ PrEP because everybody sleep with everybody. Just protect yourself.” -*Patient, Age 23, Female, Not on PrEP				
***Increase Variety and Number of PrEP Providers***					
**Context:** Culture **Innovation:** Degree of novelty, Patient experience	Increase Provider PrEP Knowledge	Providers are not PrEP informed or don’t offer PrEP (-)	Use train-the-trainer strategies Conduct educational meetings	❖ Enhance provider PrEP knowledge ❖ Inform providers about PrEP payment assistant programs	Train designated PrEP providers to help provide PrEP education to other medical providers to increase the number of providers offering PrEP in MS Share PrEP payment assistance information with providers to increase awareness of resources
**Supportive Quotes**	⇒* “At first, it was very difficult to get providers to buy into the HIV program because new programs have to prove themselves to providers. It’s because they see so many programs come and go, and so they’re always reluctant to be like, We’re gonna add this program into our routine care and practices. Is it permanent? Is this another pop-up program?”*– Staff, Clinical Quality Director, Female ⇒* “There's payment assistance, like coupons that they can add once your insurance goes through or if you don't have insurance, that covers it all, but a lot of people don't know about it.”—*Patient, Age Unknown, Female, Not on PrEP				
**Context:** Culture, Inter-organizational relationships **Innovation:** Clinical and patient experience, Degree of fit with existing practices	Increase Variety of PrEP Providers	Options for who to see to get PrEP will increase PrEP use ( +)	Conduct educational meetings Use train-the-trainer strategies	❖ Expand PrEP delivery to other specialties and types of providers ❖ Provide option to a PrEP provider not associated with their community ❖ Increase access to providers and navigators for patients to ask questions	Conduct educational meetings with clinic staff and clinicians to increase clinic staff and providers’ knowledge of PrEP Identify leaders to train providers’ in how to prescribe PrEP and increase their overall confidence prescribing PrEP. This will increase the overall number of PrEP prescriptions offered Identify specific opportunities for patients to ask questions related to PrEP
**Supportive Quotes**	⇒* “You have a certain doctor with PrEP that you go to for all your medications, and that's a main benefit, the privacy of it.”—*Patient, Age 32, Female, Not on PrEP ⇒* “Heterosexual health care providers feel like they have the right to kinda lecture you on your sexual behavior from a point of privilege. “You know you’re gay, so should be—” And it is so offensive. I would rather them be gay so they can totally understand or a very culturally competent straight man.” -*Patient, Age 28, Male, Taking PrEP				
***Enhance PrEP Provider Alliance and Trust***					
**Recipients:** Motivations, Values, & Beliefs **Innovation:** Clinical and patient experience	Enhance Patient/Provider alliance/communication	Judgement free providers increase PrEP use ( +)	Conduct educational meetings Distribute educational materials	❖ Enhance patient-provider alliance ❖ Culturally competent providers ❖ Judgement free environment ❖ Help patients weigh pros and cons ❖ Use published, scientific literature on PrEP to start the conversation ❖ Inform patients of the options for PrEP, (Truvada, Descovy, and Apretude)	Provide training to providers about cultural considerations and the different forms of PrEP (pill, injectable) and encourage providers to offer the different options tailored to patient needs to increase willingness to use PrEP Provide providers with literature on the efficacy of PrEP and the common side effects to utilize when discussing PrEP with patients in MS to increase patient understanding and willingness to use PrEP
**Supportive Quotes**	⇒* “I do [get people on PrEP], because I am transparent. And that I'm relatable. I try to always be honest with the people. I just try to tell, ‘you can't worry about what others say, but we have to do what we have to do for ourselves.’”*—Staff, Nurse Director, Female ⇒* “He explained how these were common side effects of all medicine, that the studies of bone density, they had not been proved. There was no significant difference in the bone density of people who had been on PrEP for over an extended period of time versus those who had not. This made me, way more comfortable.” -*Patient, Age 28, Male, Taking PrEP ⇒* “They listen to my questions instead of jumping in the middle, which a lotta doctors do.” -*Patient, Age 23, Female, Not on PrEP				
**Innovation:** Clinical and patient experience	Discreet Care	Concern about privacy when receiving PrEP (-)	Change service sites Develop resource sharing agreements	❖ Same reception and waiting area for all patients ❖ Provide discrete pill holders ❖ Offer secure ways to receive test results ❖ Offer on-site pharmacy or streamline pharmacy pickup	Increase the number of and variety service sites offering PrEP to help enhance confidentiality and increase comfort Collaborate with pharmacies eliminate pharmacy pickup as a barrier to PrEP uptake and persistence
**Supportive Quotes**	⇒* “We try our best to make it as private as possible. For our program, you don’t know if they’re going to just see the doctor for a regular annual checkup or a physical visit or for that program’cause it’s in one big clinic. That’s one good way that we kinda take away from the stigma.”*– Staff, Accountant, Female ⇒* “I think telemedicine, they would probably prefer that. A private area would be the next option. But I think telemedicine for those that are uncomfortable, of someone seeing them.”*—Staff, Director of Nurses, Female				
***Increase Access to PrEP***					
**Recipients:** Time & resources **Context:** Policy drivers, Inter-organizational relationships **Recipients:** Time & resources	Increase Access to PrEP	Limited access to PrEP appointments (-)	Change service sites Develop resource sharing agreements	❖ Mobile health units ❖ Home health/traveling PrEP nurse ❖ Same-day PrEP appointments ❖ Utilizing health clinics and community-based organizations ❖ Offer telemedicine appointments	Increase the number of sites delivering PrEP prescriptions to enhance access to PrEP among individuals in MS and increase PrEP use Develop partnerships with clinics and community health organizations to provide PrEP education and appointments to increase access to PrEP care Offer appointments via telemedicine to enhance adherence to PrEP appointments
**Supportive Quotes**	⇒* “It [same day PrEP appointments] would decrease the turnover rate because if you say, ‘Hey, we’re gonna get you an appointment today,’ that means you can do it today because, tomorrow, the individual may not be available, or they forgettin’ it, or change their mind.” -*Patient, Age 50, Male, Taking PrEP ⇒* “If they could do like home health or travel PrEP people, that they could come out, like they have travel nurses that do some elderly patients, or patients who aren’t local.” -*Patient, Age 28, Male, Taking PrEP ⇒* “I think if you offer a way for them to do the telemedicine, and offer ways for them to do their labs at home, we have better compliance.”*- Staff, Clinical Quality Director, Female				

### PrEP knowledge

#### Patient PrEP Awareness (i-PARIHS: Recipients)

Most patients had heard of PrEP and were somewhat familiar with the medication. One patient described her knowledge of PrEP as follows, *“I know that PrEP is I guess a program that helps people who are high-risk with sexual behaviors and that doesn't have HIV, but they're at high-risk.”-* Patient, Age 32, Female, Not on PrEP. However, many lacked knowledge of who may benefit from PrEP, where to receive a prescription, the different medications used for PrEP, and the efficacy of PrEP. Below is a comment made by a patient listing what she would need to know to consider taking PrEP. *“I would need to know the price. I would need to know the side effects. I need to know the percentage, like, is it 100 or 90 percent effective.”—*Patient, Age Unknown, Female, Not on PrEP. Patients reported learning about PrEP via television and social media commercials, medical providers, and their social networks. One patient reported learning about PrEP from her cousin. *“The only person I heard it [PrEP] from was my cousin, and she talks about it all the time, givin’ us advice and lettin’ us know that it’s a good thing.”—*Patient, Age Unknown, Female, Not on PrEP.

#### Clinic Staff PrEP Awareness (i-PARIHS: Context)

Training in who may benefit from PrEP and how to prescribe PrEP varied among clinic staff at different FQHCs. Not all clinics offered formal PrEP education for employees; however, most knew that PrEP is a tool used for HIV prevention. Staff reported learning about PrEP via different speakers and meetings. A clinic staff member reported learning about PrEP during quarterly meetings. *“Well, sometimes when we have different staff meetings, we have them quarterly, and we discuss PrEP. Throughout those meetings, they tell us a little bit of information about it, so that's how I know about PrEP.”* – Staff, Dental Assistant, Female. Some FQHC staff members reported having very little knowledge of PrEP. One staff member shared that she knew only the “bare minimum” about PrEP, stating,

*“I probably know the bare minimum about PrEP. I know a little about it [PrEP] as far as if taken the correct way, it can prevent you from gettin’ HIV. I know it [PrEP] doesn’t prevent against STDs but I know it’s a prevention method for HIV and just a healthier lifestyle.”* –Staff, Accountant, Female

A few of the organizations had PrEP navigators to which providers refer patients. These providers were well informed on who to screen for PrEP eligibility and the process for helping the patient obtain a PrEP prescription. One clinic staff member highlighted how providers must be willing to be trained in the process of prescribing PrEP and make time for patients who may benefit. Specifically, she said,

*“I have been trained [for PrEP/HIV care]. It just depends on if that’s something that you’re willing to do, they can train on what labs and stuff to order ’cause it’s a whole lot of labs. But usually, I try to do it. At least for everybody that’s high-risk.”* – Staff, OB/GYN Nurse Practitioner, Female

Another clinic staff member reported learning about PrEP while observing another staff member being training in PrEP procedures.

*“Well, they kinda explained to me what it [PrEP] is, but I was in training with the actual PrEP person, so it was kinda more so for his training. I know what PrEP is. I know the medications and I know he does a patient assistance program. If my patients have partners who are not HIV positive and wanna continue to be HIV negative, I can refer 'em.”* – Staff, Administrative Assistant, Female

### PrEP barriers

#### Barriers Receiving PrEP Services (i-PARIHS: Recipients, Innovation)

Several barriers to receiving PrEP services were identified in both patient and clinic staff interviews. There was a strong concern for the side effects of PrEP. One patient heard that PrEP could cause weight gain and nightmares, *“I’m afraid of gaining weight. I’ve heard that actual HIV medication, a lotta people have nightmares or bad dreams.” -*Patient, Age 30, Female, Not on PrEP. Another patient was concerned about perceived general side effects that many medications have. *“Probably just the [potential] side effects. You know, most of the pills have allergic reactions and side effects, dizziness, seizures, you know.” -*Patient, Age 30, Female, Not on PrEP.

The burden of remembering to take a daily pill was also mentioned as a barrier to PrEP use. One female patient explained how PrEP is something she is interested in taking; however, she would be unable to take a daily medication.

*“I’m in school now and not used to takin’ a medication every day. I was takin’ a birth control pill, but now take a shot. That was one of the main reasons that I didn’t start PrEP cause they did tell me I could get it that day. So like I wanna be in the mind state to where I’m able to mentally, in my head, take a pill every day. PrEP is somethin’ that I wanna do.” -* Patient, Age Unknown, Female, Not on PrEP

Stigma and confidentiality were also barriers to PrEP use at FQHCs. One staff member highlighted how in small communities it is difficult to go to a clinic where employees know you personally. Saying,

*“If somebody knows you’re going to talk to this specific person, they know what you’re goin’ back there for, and that could cause you to be a little hesitant in coming. So there’s always gonna be a little hesitancy or mistrust, especially in a small community. Everybody knows everybody. The people that you’re gonna see goes to church with you.” –* Staff, Accountant, Female

Some patients had a low perceived risk of HIV and felt PrEP may be an unnecessary addition to their routine. One patient shared that if she perceived she was at risk for HIV, then she would be more interested in taking PrEP, *“If it ever came up to the point where I would need it [PrEP], then yes, I would want to know more about it [PrEP].”—*Patient, Age Unknown, Female, Not on PrEP.

Some participants expressed difficulty initiating or staying on PrEP because of associated costs, transportation and/or scheduling barriers. A staff member explained how transportation may be available in the city but not available in more rural areas,

*“I guess it all depends on the person and where they are. In a city it might take a while, but at least they have the transportation compared to someone that lives in a rural area where transportation might be an issue.”* - Staff, Director of Nurses, Female

Childcare during appointments was also mentioned as a barrier, *“It looks like here a lot of people don't have transportation or reliable transportation and another thing I don't have anybody to watch my kids right now.*—Staff, Patient Navigator, Female.

#### Barriers Providing PrEP Services (i-PARIHS: Context)

Barriers to providing PrEP services were also identified. Many providers are still not trained in PrEP procedures nor feel comfortable discussing or prescribing PrEP to their patients. One patient shared an experience of going to a provider who was PrEP-uninformed and assumed his medication was to treat HIV,

*“Once I told her about it [PrEP], she [clinic provider] literally right in front of me, Googled it [PrEP], and then she was Googlin’ the medication, Descovy. I went to get a lab work, and she came back and was like, “Is this for treatment?” I was like, “Why would you automatically think it’s for treatment?” I literally told her and the nurse, “I would never come here if I lived here.” -*Patient, Age 50, Male, Taking PrEP

Also, it was reported that there is not enough variety in the kind of providers who offer PrEP (e.g., OB/GYN, primary care). Many providers such as OB/GYNs could serve as a great way to reach individuals who may benefit from PrEP; however, patients reported a lack of PrEP being discussed in annual visits. “My previous ones (OB/GYN), they’ve talked about birth control and every other method and they asked me if I wanted to get tested for HIV and any STIs, but the conversation never came up about PrEP.” -Patient, Age Unknown, Female, Not on PrEP.

### PrEP motivation

#### Motivation to take PrEP (i-PARIHS: Recipients)

Participants mentioned several motivators that enhanced patient willingness to use PrEP. Many patients reported being motivated to use PrEP to protect themselves and their partners from HIV. Additionally, participants reported wanting to take PrEP to help their community. One patient reported being motivated by both his sexuality and the rates of HIV in his area, saying, *“I mean, I'm bisexual. So, you know, anyway I can protect myself. You know, it's just bein' that the HIV number has risen. You know, that's scary. So just being, in, an area with higher incidents of cases.”—*Patient, Age Unknown, Male, Not on PrEP*.* Some participants reported that experiencing an HIV scare also motivated them to consider using PrEP. One patient acknowledged his behaviors that put him at risk and indicated that this increased his willingness to take PrEP, *“I was havin' a problem with, you know, uh, bein' promiscuous. You know? So it [PrEP] was, uh, something that I would think, would help me, if I wasn't gonna change the way I was, uh, actin' sexually.”—*Patient, Age Unknown, Male, Taking PrEP*.*

Table 3 outlines the implementation strategies identified from themes from the interview and focus group data. Below we recognize the barriers and determinants to PrEP uptake for patients attending FQHCs in MS by each i-PARIHS construct (innovation, recipient, context) [28]. Based on the data, we mapped the determinants to specific strategies from the ERIC project [26] and hypothesized the mechanism of change for each strategy [30, 31].

### Member checking focus groups

#### Sample

Two focus groups were conducted with nine staff from threeFQHCs in MS. There were six participants in the 1st focus group and three in the 2nd. Staff members were a mix of medical (e.g., nurses, patient navigators, social workers) and non-medical (e.g., administrative assistant, branding officer) personnel. Table 4 provides the demographic characteristics for the FQHC focus group participants.

**Table 4:** Demographic characteristics FQHC staff focus group participants

	*M* (*SD*)
Age	47 (11.42)
Years of experience in job role	9.16 (7.65)
	n (%)
Gender	
Male	1 (11.1%)
Female	8 (88.9%)
Race/ethnicity	
Black / African American	8 (88.9%)
American Indian or Alaska Native	1 (11.1%)
Education level	
> 1 year of college, no degree	3 (33.3%)
Associate’s degree	2 (22.2%)
Bachelor’s degree	3 (33.3%)
Master’s degree	1 (11.1%)
Role at place of employment	
Medical provider	3 (33.3%)
Non-medical staff	3 (33.3%)
Other	3 (33.3%)

Staff participating in the focus groups generally agreed that the strategies identified via the interviews were appropriate and acceptable. Focus group content helped to further clarify some of the selected strategies. Below we highlight findings by each strategy domain.

#### PrEP information dissemination

Participants specified that awareness of HIV is lower, and stigma related to PrEP is higher in rural areas. One participant specifically said,

*“There is some awareness but needs to be more awareness, especially to rural areas here in Mississippi. If you live in the major metropolitan areas there is a lot of information but when we start looking at the rural communities, there is not a lot.”* – Staff, Branding Officer, Male

Participants strongly agreed that many patients don’t realize they may benefit from PrEP and that more inclusive advertisements are needed. A nurse specifically stated,

“*When we have new clients that come in that we are trying to inform them about PrEP and I have asked them if they may have seen the commercial, especially the younger population. They will say exactly what you said, that “Oh, I thought that was for homosexuals or whatever,” and I am saying “No, it is for anyone that is at risk.”* – Staff, Nurse, Female

Further, staff agreed that younger populations should be included in PrEP efforts to alleviate stigma. Participants added that including PrEP information with other prevention methods (i.e., birth control, vaccines) is a good place to include parents and adolescents:

*“Just trying to educate them about Hepatitis and things of that nature, Herpes. I think we should also, as they are approaching 15, the same way we educate them about their cycle coming on and what to expect, it’s almost like we need to start incorporating this (PrEP education), even with different forms of birth control methods with our young ladies.”* – Staff, Nurse, Female

Participants agreed that PrEP testimonials would be helpful, specifically from people who started PrEP, stopped, and then were diagnosed with HIV. Participants indicated that this may improve PrEP uptake and persistence. One nurse stated:

*“I have seen where a patient has been on PrEP a time or two and at some point, early in the year or later part of the year, and we have seen where they’ve missed those appointments and were not consistent with their medication regimen. And we have seen those who’ve tested positive for HIV. So, if there is a way we could get one of those patients who will be willing to share their testimony, I think they can really be impactful because it’s showing that taking up preventive measures was good and then kind of being inconsistent, this is what the outcome is, unfortunately.”* – Staff, Nurse, Female

#### Increase variety and number of PrEP providers

Participants agreed that a “PrEP champion” (someone to promote PrEP and answer PrEP related questions) would be helpful, especially for providers who need more education about PrEP to feel comfortable prescribing. A patient navigator said,

*“I definitely think that a provider PrEP champion is needed in every clinic or organization that is offering PrEP. And it goes back to what we were saying about the providers not being knowledgeable on it [PrEP]. If you have a PrEP champion that already knows this information, it is gonna benefit everybody, patients, patient advocates, the provider, everyone all around. Everyone needs a champion."* – Staff, Patient Navigator, Female

Staff noted that they have walk-in appointments for PrEP available; however, they often have too many walk-in appointments to see everyone. They noted that having more resources and providers may alleviate this barrier for some patients:

*“We still have challenges with people walking in versus scheduling an appointment, but we do have same day appointments. It is just hard sometimes because the volume that we have at our clinic and the number of patients that we have that walk in on a daily basis.”* – Staff, Social Worker, Female

#### Enhance PrEP provider alliance and trust

Participants agreed that educational meetings would be beneficial and highlighted that meetings should happen regularly and emphasized a preference for in-person meetings. This is emphasized by the statement below,

*“They should be in-person with handouts. You have to kind of meet people where they are as far as learning. Giving the knowledge, obtaining the knowledge, and using it, and so you have to find a place. I definitely think that yearly in-person training to update guidelines, medication doses, different things like that."* – Staff, Patient Navigator, Female

Staff also suggested hosting one very large collaborative event to bring together all organizations that offer PrEP and HIV testing to meet and discuss additional efforts:

*“What I would like to see happen here in the state of Mississippi, because we are so high on the list for new HIV infections, I would like to see a big collaborative event. As far as PrEP goes, those that are not on PrEP, one big collaborative event with different community health centers. You do testing, we do PrEP, and the referral get split. Everyone coming together for one main purpose.”* – Staff, Patient Navigator, Female

#### Increase access to PrEP

Participants highlighted that most of the clinics they worked for already offer a variety of service sites (pharmacy, mobile clinic) but that more clinics should offer these alternative options for patients to receive PrEP. One patient navigator outlined the services they offer,

*“We have a mobile unit. We do not have a home health travel nurse. We do telephone visits. We offer primary care, OB/GYN. We have our own pharmacy. We also have samples in our pharmacy available to patients that can’t get their medicine on the same day cos we like to implement same day PrEP. It has worked for us. More people should utilize those services.”* – Staff, Patient Navigator, Female

Other staff suggested utilizing minute clinics and pharmacies at grocery stores. Highlighting, that offering PrEP at these locations may increase PrEP uptake.

## Discussion

There has been great scientific expansion of HIV prevention research and priorities must now pivot to addressing how to best implement effective interventions like PrEP [38]. PrEP remains underutilized among individuals who may benefit, particularly in Southern states such as MS [10–14]. Implementation science could help ameliorate this by identifying barriers and facilitators to PrEP rollout and uptake. We selected and defined several strategies from the ERIC project [26] to increase PrEP use utilizing FQHCs. Our results, as shown in Table 3, highlight the four domains of strategies selected: 1) PrEP Information Dissemination, 2) Increase Variety and Number of PrEP Providers, 3) Enhance PrEP Provider Alliance and Trust, and 4) Increase Access to PrEP.

### PrEP information dissemination

Firstly, individuals cannot utilize PrEP if they are not aware of its presence and utility. In Mississippi, advertising PrEP services is integral to implementation efforts given the existing stigma and lack of health literacy in this region [39]. Potential avenues for expanding PrEP awareness are integrating it into educational curriculums, adolescents’ routine preventative healthcare, and health fairs. This study compliments prior research that people should be offered sexual health and PrEP education at a younger age to increase awareness of risk, foster change in social norms and enhance willingness to seek out prevention services [40, 41]. To meet the resulting growing need for PrEP educators, healthcare professionals should receive up-to-date PrEP information and training, so that they can confidently relay information to their patients. Similar to existing research, increasing provider education could accelerate PrEP expansion [42–44]. Training programs aimed at increasing provider PrEP knowledge may increase PrEP prescriptions provided [43] by addressing one of the most frequently listed barriers to PrEP prescription among providers [45, 46].

### Increase variety and number of PrEP providers

Many patients prefer to receive PrEP at the healthcare locations they already attend and report a barrier to PrEP being limited healthcare settings that offer PrEP [39, 47–49]. The aforementioned PrEP training could increase the number of healthcare workers willing to provide PrEP services. It is also imperative that providers in a diverse range of healthcare settings (e.g., primary care, OB/GYN, pediatricians and adolescent medicine providers) join the list of those offering PrEP to reduce stigma and enhance patient comfort.

### Enhance PrEP provider alliance and trust

These results mirrored other studies in the South that have shown that using relatable healthcare providers and trusted members of the community may serve to facilitate PrEP uptake [41, 50, 51]. If patients have a larger number of PrEP providers to choose from, they can select one that best fits their needs (e.g., location, in-network) and preferences (e.g., familiarity, cultural similarities). Enhanced comfort facilitates a strong patient-provider alliance and can lead to more open/honest communication regarding HIV risk behavior.

### Increase access to PrEP

The lack of conveniently located PrEP providers is consistently reported as a structural barrier in the South [44, 52]. This creates an increase in the demand on patients to attend regular follow-up appointments. The three strategies above all play a vital role in increasing access to PrEP. If more individuals are trained to provide PrEP care, there will be more PrEP providers, and patients can choose the best option for them. A sizeable influx of new PrEP providers could help staff new care facilities and service options in the community (e.g., mobile health units, home care, community-based clinics, telemedicine). Offering PrEP via telemedicine and mobile clinics to patients has been largely supported in the literature [44, 53, 54]. Intra- and inter-organizational collaborations could similarly increase PrEP access by sharing information and resources to ensure patients get timely, reliable care.

Our results largely supported previous findings by two systematic reviews on the barriers to PrEP uptake and implementation strategies to overcome it [39, 47]. Sullivan et.al.’s review focused on the Southern U.S. [38], while Bonacci et. al. explored steps to improve PrEP equity for Black and Hispanic/Latino communities [47]. Both agreed that barriers to PrEP access are complex. Thus, cooperation from policymakers and the expansion of state Medicaid or targeted Medicaid waivers is vital to make PrEP attainable for those living in the coverage gap. Further, many FQHCs receive Ryan White funding for HIV care and treatment, contracting flexibility in the utility of these other sources of support may aid in eliminating the cost of PrEP as a barrier. They also stressed the need for educating community members and healthcare personnel about PrEP, increasing and diversifying PrEP service sites, normalizing PrEP campaigns and screening to alleviate stigma, and streamlining clinical procedures to facilitate the option for same-day PrEP. However, they also noted that these strategies are easier said than done. This further highlights the need for prioritizing research efforts towards implementation studies for effectiveness and practicality of overcoming the complex and systemic needs around HIV prevention/treatment.

The present study was able to build on past findings by providing a more holistic view of the barriers to PrEP use and possible strategies to address them through querying PrEP-eligible patients, medical providers, and non-medical staff. By interviewing a diverse range of stakeholders, it was possible to identify unmet patient needs, current PrEP care procedures and infrastructure, and attitudes and needed resources among those who could potentially be trained to provide PrEP in the future.

#### Limitations

Our results are limited to participants and clinic staff who were willing to engage in a research interview to discuss PrEP and FQHCs. Results are only generalizable to Mississippi and may be less relevant for other geographic areas. However, this is a strength given these strategies are meant to be tailored specifically to FQHCs in MS. Due to COVID-19 restrictions, interviews were conducted via Zoom. This allowed us to reach participants unable to come in physically for an interview and may have increased their comfort responding to questions [55]. However, some participants may have been less comfortable discussing via Zoom, which may have limited their willingness to respond.

## Conclusions

This study highlighted the need for implementing PrEP strategies to combat HIV in Mississippi. PrEP knowledge, barriers, and motivation were identified as key factors influencing PrEP utilization, and four domains of strategies were identified for improving PrEP accessibility and uptake. Future research should further refine and assess the feasibility and acceptability of selected and defined implementation strategies and test strategies.

## Data Availability

De-identified data from this study are not available in a public archive due to sensitive nature of the data. De-identified data from this study will be made available (as allowable according to institutional IRB standards) by emailing the corresponding author.

## Abbreviations

MS: Mississippi
PrEP: Pre-Exposure Prophylaxis
FQHCs: Federally Qualified Health Centers
i-PARIHS: Integrated-Promoting Action on Research Implementation in Health Services
ERIC: Expert Recommendations for Implementing Change
MSM: Men Who Have Sex With Men
